# Acquisition of Full-Length Viral Helicase Domains by Insect Retrotransposon-Encoded Polypeptides

**DOI:** 10.3389/fmicb.2015.01447

**Published:** 2015-12-22

**Authors:** Ekaterina Lazareva, Alexander Lezzhov, Nikita Vassetzky, Andrey Solovyev, Sergey Morozov

**Affiliations:** ^1^Department of Virology, Faculty of Biology, Lomonosov Moscow State UniversityMoscow, Russia; ^2^Laboratory of Eukaryotic Genome Evolution, Engelhardt Institute of Molecular Biology, Russian Academy of SciencesMoscow, Russia; ^3^Genetic Engineering of Plant Viruses, A.N. Belozersky Institute of Physico-Chemical Biology, Moscow State UniversityMoscow, Russia

**Keywords:** plant virus, helicase, retrotransposons, RNA silencing, RNA silencing suppressor

## Abstract

Recent metagenomic studies in insects identified many sequences unexpectedly closely related to plant virus genes. Here we describe a new example of this kind, insect R1 LINEs with an additional C-terminal domain in their open reading frame 2. This domain is similar to NTPase/helicase (SF1H) domains, which are found in replicative proteins encoded by plant viruses of the genus Tobamovirus. We hypothesize that the SF1H domain could be acquired by LINEs, directly or indirectly, upon insect feeding on virus-infected plants. Possible functions of this domain in LINE transposition and involvement in LINEs counteraction the silencing-based cell defense against retrotransposons are discussed.

## Introduction

RNA helicases are RNA-binding proteins that utilize the energy derived from the hydrolysis of NTPs to unwind double-stranded (ds) nucleic acids. They are classified into six superfamilies designated SF1H to SF6H on the basis of conserved helicase motifs (Gorbalenya et al., 1989; Koonin and Dolja, 1993; Jankowsky and Fairman, 2007; Singleton et al., 2007). Positive-stranded RNA viruses have been reported to encode their own replicative RNA helicases which represent SF1H, SF2H, and SF3H proteins (Koonin and Dolja, 1993; Kadaré and Haenni, 1997). Helicases are encoded by all plus-RNA plant and animal viruses with the genomes that are larger than 7 kb (Koonin and Dolja, 2014). Their main function in energy-dependent unwinding of ds nucleic acid regions during replication by melting intra-molecular secondary structures or by separating RNA duplexes including viral plus and minus strands. In addition, viral RNA helicases are believed to play important roles in many processes involving RNA molecules. Particularly, several accompanying enzymatic activities and functions were reported for helicases, such as RNA triphosphatase, energy-independent RNA chaperone, and stripping proteins from the viral RNA (RNPase) (Jankowsky et al., 2001; Rajkowitsch et al., 2007; Gebhard et al., 2012; Leitão et al., 2015).

Recently, we hypothesized that gaining a new silencing suppression function by a SF1H domain of virus replicative protein could precede its duplication in the context of the same viral genome or a horizontal transfer to a foreign virus genome. These events may result in evolving a specialized “accessory” helicase possessing the activities of a viral silencing suppressor (VSR) and movement protein (Morozov and Solovyev, 2012, 2015).

The genomes of animals, insects, plants, and fungi were found to carry a number of virus-like sequences including helicase-coding fragments that are closely or distantly related to the present-day positive stranded RNA viruses (Katzourakis and Gifford, 2010; Holmes, 2011; Cui and Holmes, 2012; Feschotte and Gilbert, 2012; Kondo et al., 2013). Additionally, our observations show that cucumoviral and pomoviral helicase-encoding sequences, which can be transcribed into hairpin RNA structures and potentially confer virus resistance, exist in genomes of plants (to be published elsewhere; see also Tromas et al., 2014). Moreover, recent studies revealed occurrence of tobamo-like movement protein sequences in plant genomes (Mushegian and Elena, 2015). In insects, metagenomic studies identified many virus-like sequences including helicase-encoding genes, most of which were unexpectedly close to those encoded by plant viruses, particularly, to SF1H domains in the family Virgaviridae (Adams et al., 2009; Cui and Holmes, 2012; Cook et al., 2013). Frequent interactions between plants and insects, for example, through pollination and feeding, and the insect origin of many plant viruses can explain occasional invasion of insect genomes by plant virus sequences (Cui and Holmes, 2012), which occurs as a result of horizontal gene transfer, the process responsible for the transfer of genes between viruses as well as from viruses to cellular genomes.

In view of these observations, we analyzed available insect sequences in an attempt to identify new “accessory” helicases showing sequence similarity to replicative SF1H domains encoded by plant viruses.

## Occurrence of SF1H-coding sequences in insect retrotransposons

Using replicative SF1H sequences of tobamoviruses *Tomato mosaic virus* (ToMV, GeneBank accession AJ132845) and *Turnip vein-clearing virus* (Z29370) as queries for TBLASTN search at NCBI, we found two significant matches to the encoded amino acid sequences in the transcriptome shotgun assembly databases of *Heliconius melpomene* and *Ostrinia nubilalis* (for GeneBank accession numbers, see Supplementary Materials and Methods hereafter). Amazingly, our further analysis revealed more than 40 significant matches to the encoded amino acid sequences in the whole-genome shotgun assemblies of *Plutella xylostella* chromosomes (data not shown). The genome-integrated SF1H sequences share a similar level of amino acid sequence identity with different tobamoviruses. In particular, ToMV SF1H had 36% identity (*E*-value = 1e-24) with *P. xylostella*, 30% identity with *H. melpomene* (*E*-value = 6e-23), and 32% identity (*E*-value = 3e-25) with *O. nubilalis* sequences.

When a consensus sequence based on an alignment of three tobamoviral-like SF1H domains encoded by *H. melpomene, O. nubilalis*, and *P. xylostella* was used as a query for TBLASTN search of virus sequence database, the most significant similarity of the consensus sequence was found to tobamoviral and related NTPase/helicase replicative domains of other plant viruses of the family Virgaviridae.

**Figure 1:**
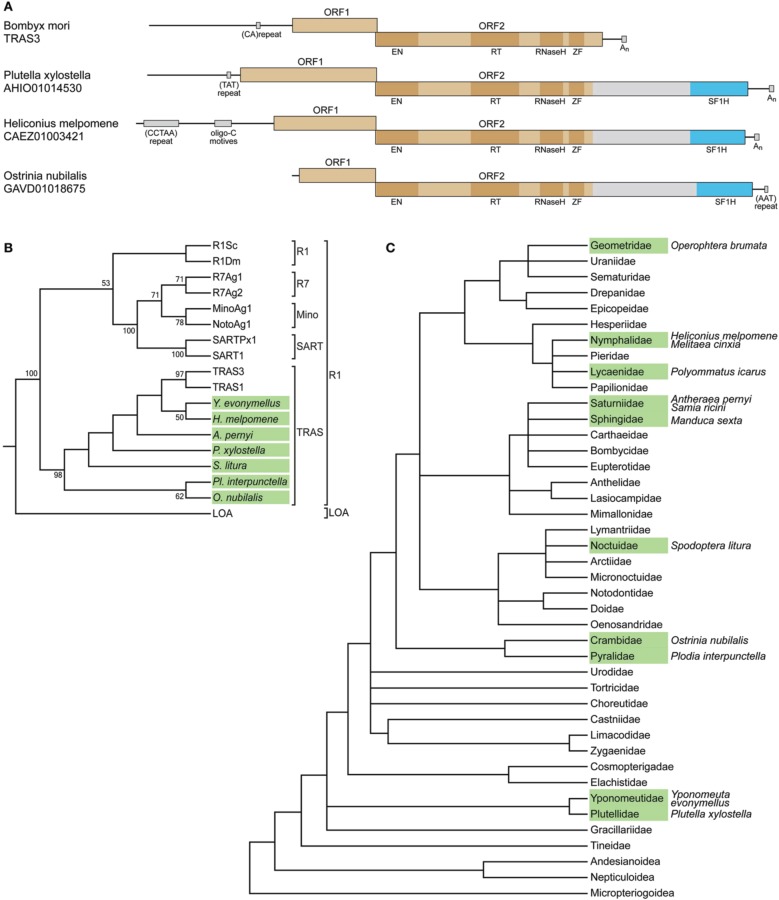
**SF1H domains in insect genomes. (A)** SF1H domains encoded by insect LINEs. Organization of three insect LINEs encoding tobamovirus-like SF1H domains and closely related *Bombyx mori* LINE TRAS3. Boxes schematically represent open reading frames ORF1 and ORF2. Blue boxes represent the tobamovirus-like SF1H domains. Functional domains in ORF2 are indicated by dark boxes. EN, endonuclease domain; RT, reverse transcriptase domain; ZF, zinc finger domain. Conserved DNA sequence signatures outside ORF1/ORF2 region are indicated by small boxes. **(B)** SF1H-encoding LINEs belong to the TRAS superfamily of the R1 clade of LINEs. The phylogenetic tree is based on RT domain amino acid sequence alignment generated for SF1H-encoding LINEs and other LINEs. Seven species with SF1H-encoding LINEs, for which RT domain sequences are available, were included into the analysis. Insect species with SF1H-encoding LINEs are show in green. Conventional names for previously known LINEs are given. Clades R1 and LOA are indicated on the right. Superfamilies within the R1 clade are shown. Only bootstrap values ≥50% are shown. **(C)** Occurrence of SF1H-encoding LINEs in different families of Lepidoptera. The tree represents the synoptic view of the phylogeny of major Lepidoptera taxa (Wheeler et al., 2013). Families for which SF1H-encoding LINEs were identified are shown in green. Species with SF1H-encoding LINEs are indicated on the right.

The identified *H. melpomene, O. nubilalis*, and *P. xylostella* sequence regions encoding the tobamovirus-like SF1H domains were found within long interspersed elements (LINEs). LINEs represent a class of non-LTR (long terminal repeat) retrotransposons widely spread in eukaryotic genomes. The majority of functional LINEs code for ORF1 and ORF2 proteins, the latter comprises several functional domains, which specify retropositional activities such as reverse transcriptase, endonuclease and, in some cases, RNase H (Eickbush and Jamburuthugoda, 2008). In the LINEs of *H. melpomene, O. nubilalis*, and *P. xylostella*, the tobamovirus-related SF1H sequence was encoded as an additional domain at the C-terminus of ORF2-encoded protein (Figure 1A), which has also the complete array of functional domains typical for similar LINEs lacking the SF1H domain, such as *Bombyx mori* TRAS3 (see below). The most C-terminal region in the LINE ORF2 is known to tolerate insertions of foreign protein domains. For example, a family of plant LINEs was found to code for the ORF2 protein with an additional C-terminal RNase H domain of archaeal origin; moreover, this domain appeared autonomously active (Smyshlyaev et al., 2013). It should be noted that the SF1H-like domain in the ORF2 protein is separated from the preceding LINE-specific sequence by a spacer of more than 300 amino acids showing no significant similarity to viral or non-viral sequences in all available databases (Figure 1A).

The three insect species for which SF1H-like domain-encoding LINEs were found, *H. melpomene, O. nubilalis*, and *P. xylostella*, belong to different families of the order Lepidoptera (moths and butterflies). We further analyzed whether such LINEs could be found in other Lepidoptera families and other insect orders. Nucleotide and amino acid consensus sequences of SF1H based on the alignments of *H. melpomene, O. nubilalis*, and *P. xylostella* were used as queries for searches of nucleotide and translated nucleotide databases, respectively.

All genomic sequences available for the order Lepidoptera were analyzed as well as the well-represented EST (expressed sequence tag) databases. In addition to the three initially identified species, the SF1H-like domain-encoding LINEs were found in nine other species, namely, *Operophtera brumata* (the family Geometridae), *Polyommatus icarus* (Lycaenidae), *Samia ricini* (Saturniidae), *Antheraea pernyi* (Saturniidae), *Manduca sexta* (Sphingidae), *Spodoptera litura* (Noctuidae), *Melitaea cinxia* (Nymphalidae), *Plodia interpunctella* (Pyralidae), and *Yponomeuta evonymellus* (Yponomeutidae) (Figure 1C; Supplementary Table S1). Importantly, SF1H-like domain-encoding LINEs were identified not in all analyzed insect families. This could be due to a low coverage of the genomes in those particular species. However, for two species with completely sequenced genomes, *B. mori* (Bombycidae) and *Danaus plexippus* (Nymphalidae), no SF1H-like domain-encoding LINEs were found. This finding indicated that not all lepidopterans contain LINEs of this type. On the other hand, the presence of these LINEs in different branches of the Lepidoptera phylogenetic tree including early diverged families Plutellidae and Yponomeutidae (Figure 1C) demonstrate the wide occurrence of SF1H-like domain-encoding LINEs in this insect order.

Further, we analyzed species of three orders most closely related to Lepidoptera. Overall, for the order Hymenoptera (wasps, bees, and ants) genomic sequences were analyzed in 57 species, for 27 of which the complete or very extensive genome information was available; for the order Diptera (flies), for 32 species (13 with complete or well-represented genomes); and for the order Trichoptera (caddisflies), for four species (Supplementary Table S1). Again, well-represented EST databases were analyzed whenever possible. As a result, no SF1H-like domain-encoding LINEs were found in these insect orders. Therefore, we conclude that the LINEs of the R1 clade carrying SF1H-like domains in their ORF2 occur in at least nine families of the order Lepidoptera but not in other related orders.

## Evolution of retrotransposons encoding SFI helicase domains

The phylogeny of LINEs is conventionally based on their RT domain sequences (Malik et al., 1999). To date, phylogenetic analysis revealed 21 phylogenetic groups, or clades, of LINEs (Malik et al., 1999; Kojima and Fujiwara, 2005; Novikova et al., 2007). The analysis of proteins encoded by LINEs of different clades made it possible to propose a probable evolutionary scenario for LINE structural organization involving sequential acquisition of functional domains (Malik et al., 1999). Therefore, acquisition of new domains located at the C-terminus of the ORF2 protein in plant LINEs (Smyshlyaev et al., 2013) and insect LINEs (this paper) are in agreement with the generally accepted concept of LINE evolution.

To analyze the relation of the SF1H-encoding LINEs to other LINEs, a multiple amino acid alignment of RT sequences was generated. A maximum-likelihood tree demonstrated a close relationship of the SF1H-encoding LINEs to TRAS1 and TRAS3 (Figure 1B) and established that these elements belong to the TRAS superfamily of the R1 clade of LINEs (Kojima and Fujiwara, 2003; Lavoie et al., 2013).

Since the R1 clade LINEs were described in arthropods but not plants (Malik et al., 1999), we hypothesize that the SF1H domain could be acquired by LINEs upon insect feeding on virus-infected plants. The transfer of SF1H sequence from a plant virus genome to an ancestral insect LINE unlikely was a direct process of their recombination occurred via, for example, a template switch during reverse transcription. Taking into account that such an event, if has led to the inheritance of SF1H-encoding LINEs, should take place in germline cells, one can presume that the plant virus SF1H domain could be initially transferred to the genome of an insect virus capable of infecting germline cells.

In the phylogenetic tree of Lepidoptera, the identified SF1H-encoding LINEs are found in several distant lineages including the superfamily Yponomeutoidea (families Plutellidae and Yponomeutidae) diverged early in the Lepidoptera evolution, indicating that the SF1H domain could be acquired by LINEs in an ancestor that existed before the divergence of Lepidoptera lineages (Figure 1C). On the other hand, such elements are not found in the completely sequenced genome of *Bombix mori* (the family Bombycidae) but identified in representatives of the families Saturniidae and Sphingidae, which, together with Bombycidae, belong to the superfamily Bombycoidea (Figure 1C). Even more surprisingly, in the family Nymphalidae, the SF1H-containing LINEs are found in *H. melpomene*, but not in the *D. plexippus* genome, for which the complete sequence is available. These observations show that the SF1H-containing LINEs demonstrate a “mosaic” distribution among genomes of the phylogenetically related taxa of Lepidoptera.

Generally, three different evolutionary mechanisms can account for the observed mosaic distribution of SF1H-containing LINEs. First, these elements could evolve in a progenitor of present-day Lepidoptera and inherited vertically, being further removed from the genome in some lineages. Indeed, retrotransposons in *Drosophila* are known to be subject of frequent transposition and elimination by selection (Eickbush and Furano, 2002). Although not known in Lepidoptera, this exemplifies transposon removal in insects. Second, plant-virus-like SF1H domains could be independently acquired by LINEs of distant Lepidoptera species. Third, SF1H-containing LINEs could be transmitted between Lepidoptera species by horizontal transfer, which is well documented for DNA transposons and also reported, although as a rare event, for LINEs (Sánchez-Gracia et al., 2005; Novikova et al., 2007; Sormacheva et al., 2012; Lavoie et al., 2013). To evaluate the involvement of horizontal transfer in the distribution of SF1H-containing LINEs, we carried out the analysis of evolutionary rates developed by Malik et al. (1999). Amino acid divergence values calculated for the RT domain of SF1H-containing LINEs of *P. xylostella* (family Plutellidae diverged early in the Lepidoptera evolution) and several species of more recently diverged families were found to be in the range expected for vertically inherited retrotransposons diverged from a last common ancestor at the estimated time of Plutellidae divergence from other analyzed families (Supplementary Figure S1). These observations support the vertical inheritance of SF1H-containing LINEs and their elimination from the genomes of some present Lepidoptera families.

On the other hand, the *P. xylostella* genome contains a higher, compared to other species, number of SF1H-encoding LINEs (Supplementary Table S1), demonstrating that while in some evolutionary lineages these elements were eliminated, in others they underwent a transpositional burst.

## Possible functions of virus SF1H helicases encoded by insect retrotransposons

What functional/evolutionary benefits could be gained by retrotransposons from the acquired SF1H domains? Speculatively, the expression of LINE-encoded SF1H domains may target the following processes: first, the nucleic acid synthesis during retrotransposition due to the ability of SF1H to unwind duplexes and work as RNPase; second, the RNA chaperone activity during the recognition of the primary transposon transcript by reverse transcriptase complex before starting the complementary DNA synthesis, or by RNase H at the later steps of transposition. Indeed, yeast RNA helicases are involved in retrotransposition of LTR-containing element Ty3 (Bilanchone et al., 2015). Additionally, the replicative SF1H domain of plant tobamoviruses has the VSR function (Csorba et al., 2007; Wang et al., 2012), which could be retained in SF1H domains encoded by insect LINEs. In fact, plant, insect, and animal VSRs are known to be functional in heterologous eukaryotic model systems (Berry et al., 2009; Jing et al., 2011; Maliogka et al., 2012; Zhu et al., 2012).

Retrotransposons are controlled by the RNA interference mechanisms at both the transcriptional and post-transcriptional levels (Ito, 2012; Peng and Lin, 2013; Ross et al., 2014). For example, TRAS1 LINE, which is closely related to the SF1H-encoding LINEs (Figure 1B), is shown to be a target of piRNA regulation: TRAS1 transcription is significantly increased when piRNA pathway is compromised in insect cells (Tatsuke et al., 2010). One can speculate that SF1H can be advantageous for LINEs, if it is capable of silencing suppression in insect cells and inhibits the formation of functional LINE-targeted silencing effector complexes either by sequestering LINE-specific small RNAs, or by preventing their biogenesis. This assumption does not seem unlikely considering that a plant virus VSR has been shown to impair the endogenous siRNA-mediated retrotransposon silencing in the heads and ovaries of *D. melanogaster* (Berry et al., 2009).

**Figure 2:**
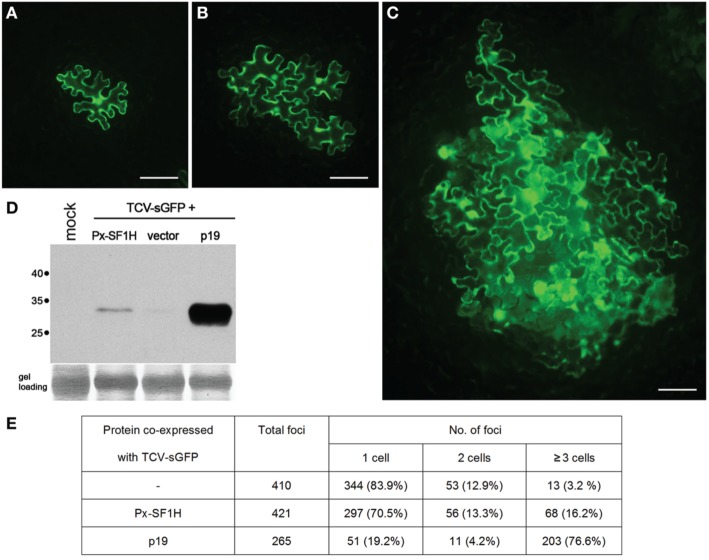
**Silencing suppression activity of SF1H domain encoded by *P. xylostella* LINE (Px-SF1H)**. For detection of possible VSR activity of Px-SF1H, an assay based on complementation of Turnip crinkle virus (TCV) cell-to-cell movement in plants (Powers et al., 2008) was used. The TCV capsid protein (CP), a well characterized VSR, is required for virus cell-to-cell transport. A TCV derivative with the CP gene replaced with the synthetic GFP gene (TCV-sGFP), being incapable of antiviral silencing suppression, is confined to primary infected cells. Since the TCV-sGFP movement ability is shown to be restored by VSRs provided in trans, the silencing suppression function of a protein can by identified by its ability to complement the TCV-sGFP cell-to-cell transport, which is visualized by GFP fluorescence in virus-infected cells. *Nicotiana benthamiana* leaves were infiltrated with a culture of *Agrobacterium tumefaciens* carrying PZP-TCV-sGFP, binary vector with cloned TCV-sGFP genome (Powers et al., 2008) mixed with an agrobacterial cultures expressing either Px-SF1H or TBSV p19, a potent VSR used as a positive control. As a negative control, TCV-sGFP was co-infiltrated with a culture carrying an empty binary vector. To ensure that TCV-sGFP is expressed in individual leaf cells located at large distances from each other, TCV-sGFP agrobacterial culture was 1250-fold diluted prior to leaf infiltration, while Px-SF1H, p19, and the vector control were infiltrated at a high density of bacterial culture resulting in protein expression in most, if not all, cells in the infiltrated leaf area. Fluorescent microscopy of agroinfiltrated leaves was carried out 5 days after infiltration (dpi). In all cases, as expected, the TCV-GFP infection was initiated in cells located both in the leaf epidermis and the underlying mesophyll. As internal cell layers, due to leaf thickness, could not be observed in focus under a microscope, the size of infection foci initiated in the epidermis was analyzed. The fluorescent foci consisting of one, two, or three and more epidermal cells were counted. **(A–C)** representative fluorescent microscopy images of infection foci consisting of TCV-sGFP-infected cells expressing GFP. Scale bars, 100 μm. **(A)** single-cell TCV-sGFP infection locus. **(B)** four-cell locus imaged for co-expression of TCV-sGFP and Px-SF1H. **(C)** large infection locus imaged for co-expression of TCV-sGFP and p19. **(D)** To include in the analysis the infection foci initiated in cells located in the leaf mesophyll, infiltrated leaf samples collected at 5 dpi were analyzed by Western blotting with GFP-specific antibodies. One lane in the gel represents a pooled sample containing discs of equal weight from three individual leaves infiltrated with a particular combination of constructs. Mock, a negative control from buffer-infiltrated leaves; vector, a control co-infiltration of TCV-sGFP with an empty binary vector. Positions of molecular weight markers are shown on the left. **(E)** The size of foci formed by TCV-sGFP co-expressed with Px-SF1H, p19, or empty vector. In the control co-infiltration of TCV-sGFP and empty vector, the GFP fluorescence was found predominantly in individual cells. As anticipated, co-infiltration of TCV-sGFP and p19 dramatically increased the size of infection foci. Co-infiltration of TCV-sGFP and Px-SF1H resulted in moderate increase in the focus size. Taking the number of the three-cells loci as an indication of viral transport, we conclude that Px-SF1H is able to promote cell-to-cell movement of TCV-sGFP.

This hypothesis finds some support in our experiments, in which the *P. xylostella* LINE-encoded SF1H domain complemented cell-to-cell transport of a VSR-deficient plant virus (Figure 2). Such complementation was suggested to be indicative of the VSR activity (Powers et al., 2008; Lukhovitskaya et al., 2013, 2014); however, it should not be considered as an unequivocal demonstration of the VSR function of the tested protein (Shi et al., 2009). Nevertheless, these preliminary observations show that LINE-encoded SF1H domain can indeed retain the VSR function, and its further evaluation will be an important line of future research, which employs other experimental systems including analysis of the SF1H VSR activity in insect cells. Since, according to the *P. xylostella* transcriptome database (http://iae.fafu.edu.cn/DBM/), the mRNA coding for the SF1H domain of SF1H-containing LINE was found among expressed genes at least in the egg, late larval, and pupal stages, future experiments on heterologous expression of native and mutant SF1H LINEs can shed light on possible influence of the SF1H domain on LINE mRNA synthesis and stability. Another experimental line can include investigation of the helicase activity of LINE-encoded SF1H domain on different substrates, primarily LINE replication intermediates.

## Concluding remarks

Considering the future directions of the experimental elucidation of the significance of viral helicase domains acquired by retrotransposons, we envisage studies of functional properties and expression of retrotransposon-encoded SF1H domains in insect organisms and cultured insect cells. However, it should be stressed that the data presented in this paper can represent only the tip of the iceberg, since preliminary database searches reveal multiple occurrences of so-far uncharacterized SF1H-like sequences in the genomes of non-lepidoteran insects. Additionally, cucumoviral and pomoviral helicase sequences were revealed in close proximity to plant LTR-type retrotransposons. Therefore, further extensive bioinformatics analysis can shed light on the whole picture of viral SF1H invasion of eukaryotic genomes, while functional studies of LINE-encoded SF1H domains will clarify their functional significance in the context of insect retrotransposons.

## Author contributions

AS and SM designed and planned the research. EL and AL performed the experiments. AS and SM performed database searches. NV performed phylogenetic analysis. AS, NV, and SM wrote the manuscript. All authors reviewed the manuscript.

## Funding

Sequence bank mining and phylogenetic analysis was performed by NV at the Engelhardt Institute of Molecular Biology with support of the Russian Foundation for Basic Research (grant 13-04-01678). All other work was performed by EL, AL, AS, and SM at Moscow State University with financial support of the Russian Science Foundation (grant 14-14-00053).

### Conflict of interest statement

The authors declare that the research was conducted in the absence of any commercial or financial relationships that could be construed as a potential conflict of interest.
