# Supplementary material for: Acquisition of Full-Length Viral Helicase Domains by Insect Retrotransposon-Encoded Polypeptides
Source: Front Microbiol. 2015 Dec 22;6:1447. doi: 10.3389/fmicb.2015.01447 (PMC4686597; doi:10.3389/fmicb.2015.01447)
Supplement: Supplementary file 1 [file Table1.DOC]

**Supplementary Materials and Methods**

Database searches and sequence analysis

The initial findings were obtained by BLAST search at the NCBI website (http://blast.ncbi.nlm.nih.gov/Blast.cgi). The database mining in the genomes and transcriptomes of insects was performed using the Smith-Waterman (for DNA queries) and the FASTY (for protein queries) tools within the FASTA package (Pearson and Lipman, 1988). We used nucleotide and amino acid consensus sequences generated from the alignment of the SF1H domain (*Viral_helicase1* in Pfam) from *H. melpomene* (CAEZ01003421), *O. nubilalis* (GAVD01018675), and *P. xylostella* (AHIO01014530) as queries. Genomic banks of insects were extracted from different sources shown in Supplementary Table S1. The hits were analyzed by Pfam (Finn et al., 2014; http://pfam.xfam.org/) to find other domains of LINE ORF2 (namely, *Exo_endo_phos_2*, *RVT_1*, and *RNase_H*) upstream of the SF1H domain. The sequence ‘*RVT_1…* *RNase_H…Viral_helicase1*’ (or even ‘*RNase_H…Viral_helicase1*’ in a few cases) was considered as a SF1H-containing LINE.

Multiple sequence alignments were generated using the CLUSTAL W program (Thompson et al., 1994) and manually corrected by GeneDoc (http://www.nrbsc.org/gfx/genedoc/ebinet.htm). Phylogenetic trees were inferred using the maximum-likelihood method implemented in the PHYLIP package (http://evolution.genetics.washington.edu/phylip/). The alignment used for the tree of LINEs included amino acid sequences of the reverse transcriptase domain (*RVT_1*) with truncated ~120 amino acids at the N-terminus since only such sequence was available for *Y. evonymellus* (the topology of tree based on the full-length sequences without *Y. evonymellus* was the same; data not shown). LOA sequence was used as the outgroup.

Accession numbers for GeneBank entries containing the sequences of SF1H-encoding LINEs, which were used for phylogenetic analysis based on RT sequences, are as follows. *Plutella xylostella*, AHIO01014530; *Heliconius melpomene*, CAEZ01003421; *Ostrinia nubilalis*, GAVD01018675; *Yponomeuta evonymellus*, GASG01123211; *Antheraea pernyi*, GBZF01003318; *Spodoptera litura*, GBBY01001816; and *Plodia interpunctella*, LN813111.

Recombinant clones

The TBSV p19 cDNA was obtained by RT-PCR of total RNA from TBSV-infected plants with primers 5’-CAATAAACCATGGAACGAGCTATAC and 5’-AGAGTCTAGATTACTCGCTTTCTTTTTCG digested with *Nco*I and *Xba*I and cloned into similarly digested binary vector pLH*described before (Solovyev et al., 2013). For cloning of *P. xylostella* LINE-encoded helicase domain (accession no. AHIO01014530, positions 14111-15117), the respective gene fragment was amplified with the specific primers Px-P (5’-GCGAATTCCAGGATTATGCTAGAGGACAAAATC) and Px-M (5’-GCTCTAGACTATTCCGCTCCATTTTGCGTTGC) from DNA isolated from *P. xylostella* larvae and cloned into the pBluescript vector as a *Eco*RI-*Xba*I-fragment. After removing the internal *Nco*I site by overlap PCR, the gene was amplified with the primers 5’-CGAGCCATGGTTGAGGACAAAATCCTAGCAAAAGC and Px-M. The product was digested with *Nco*I and *Xba*I and cloned into pLH*.

Plant agroinfiltration and analysis

Agroinfiltration of *N. benthamiana* leaves was carried out as described previously^60^. Prior to infiltration, agrobacterial cultures were adjusted to a final density of OD_600_=0.5 for PxHEL, p19, and empty pLH* vector; and OD_600_=0.0004 for PZP-TCV-sGFP. Then the PZP-TCV-sGFP culture was mixed with an equal volume of PxHEL, p19, or pLH* culture. Infiltrated leaves were examined using a Zeiss Axiovert 200M microscope, and cell images were captured with a Hamamatsu C4742 camera. Western blotting was carried out with anti-GFP antibody conjugated to horseradish peroxidase (Rockland) and the ECL kit (GE Healthcare).

**Supplementary Fig. S1**


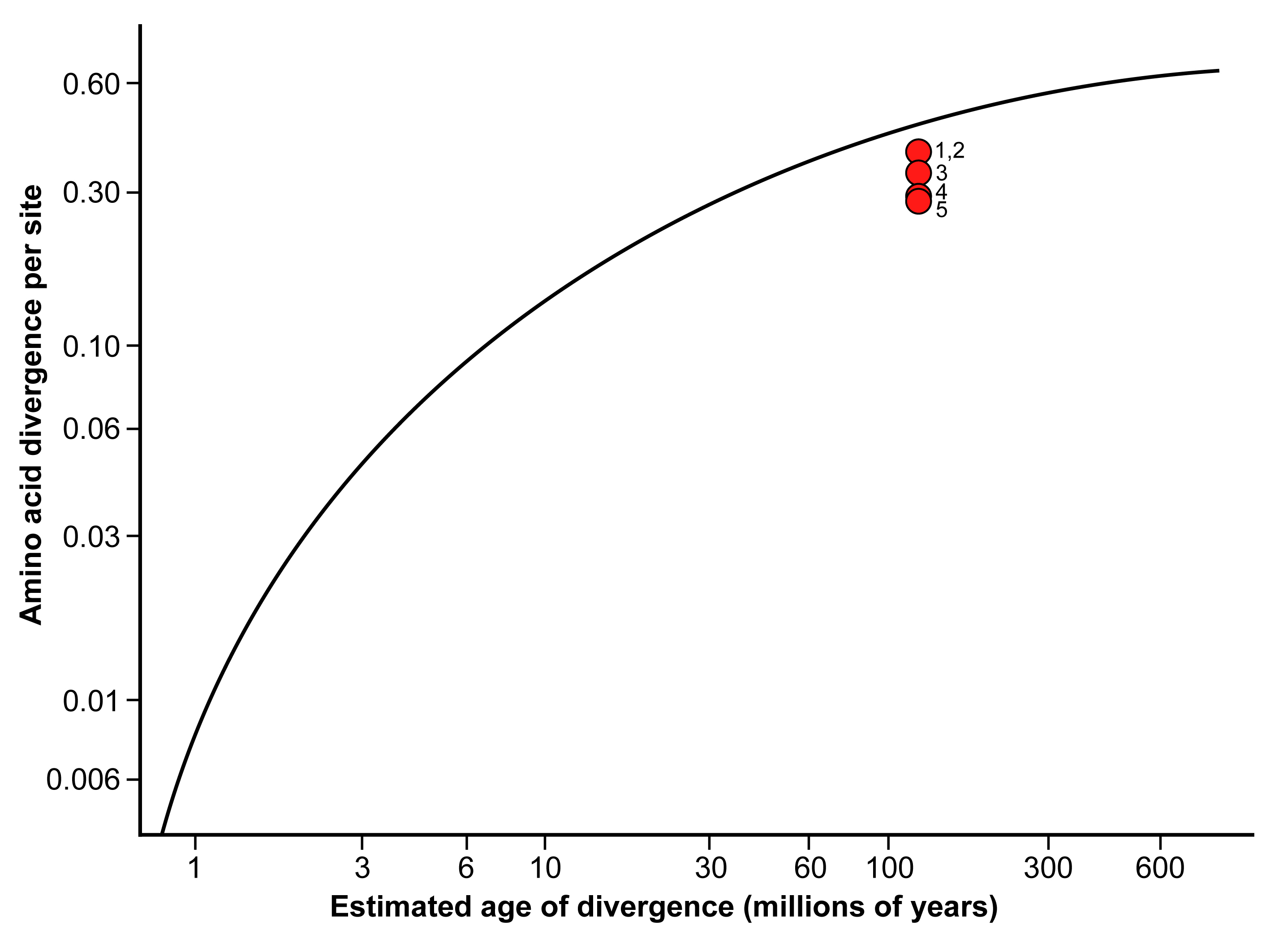


Supplementary Fig. S1. Divergence-versus-age analysis of SF1H-encoding LINEs.

Amino acid divergence per site was calculated for the sequences of complete RT domain (Malik et al., 1999). The curve for arthropod non-LTR retrotransposons is reproduced from Malik et al. (1999). Amino acid divergence values were calculated for LINEs of *P. xylostella* (family Plutellidae diverged early in the Lepidoptera evolution) versus several species of more recently diverged families. The estimated time of Plutellidae divergence from other analyzed families is 117 millions of years (Wahlberg et al., 2013). Divergence calculations are shown for pairwise sequence comparisons of the *P. xylostella* RT domain with those of *H. melpomene* (1), *P. interpunctella* (2), *A. pernyi* (3), *S. litura* (4) and *O. nubilalis* (5).

**Supplementary Table S1.**

Search for SF1H-encoding LINEs in Lepidoptera (butterflies and moths) and three most closely related orders: Trichoptera (caddisflies), Diptera (flies), and Hymenoptera (wasps, bees, and ants).

| **Order** | **Suborder** | **Superfamily** | **Family** | **Subfamily** | **Species** | **Sequence source** | | **Bank size, kb** | **RT domain hits** | **SF1H domain  hits** | **SF1H-LINE domain pattern** |
| --- | --- | --- | --- | --- | --- | --- | --- | --- | --- | --- | --- |
|  |  |  |  |  |  |  |  |  | **DNA vs DNA /  protein vs DNA** | |  |
| Lepidoptera | Glossata | Eriocranioidea | Eriocraniidae |  | *Dyseriocrania subpurpurella* | GenBank | | 51,115 | 2 / 3 | 0 / 0 |  |
|  |  | Hepialoidea | Hepialidae |  | *Triodia sylvina* | GenBank | | 76,701 | 5 / 0 | 0 / 0 |  |
|  |  | Castnioidea | Castniidae |  | *Telchin licus* | GenBank | | 15,060 | 0 / 0 | 0 / 1 | 0 |
|  |  | Zygaenoidea | Zygaenidae |  | *Zygaena fausta* | GenBank | | 46,849 | 4 / 14 | 0 / 3 |  |
|  |  | all other species in the upper four superfamilies | | | | GenBank | | 5,028 | 0 / 0 | 0 / 0 |  |
|  |  | Gelechioidea |  |  | all species | GenBank | | 17,393 | 0 / 0 | 0 / 0 |  |
|  |  | Bombycoidea | Saturniidae |  | *Actias selene* | GenBank | | 31,746 | 2 / 7 | 0 / 1 | 0 |
|  |  |  |  |  | *Antheraea assama* | GenBank | DNA | 23,473 | 2 / 4 | 0 / 0 |  |
|  |  |  |  |  |  |  | EST | 16,581 | 0 / 0 | 0 / 0 |  |
|  |  |  |  |  | *Antheraea pernyi* | GenBank | | 32,319 | 6 / 10 | 1 / 1 | 1 |
|  |  |  |  |  | *Antheraea yamamai* | GenBank | | 29,687 | 4 / 9 | 5 / 8 | 0 |
|  |  |  |  |  | *Rhodinia newara* | GenBank | | 32,862 | 8 / 12 | 1 / 1 | 0 |
|  |  |  |  |  | *Samia cynthia* | WildSilkbase | EST | 9,285 | 0 / 0 | 0 / 0 |  |
|  |  |  |  |  | *Samia ricini* | GenBank | | 29,896 | 1 / 2 | 3 / 4 | 1 |
|  |  |  | Bombycidae |  | *Bombyx mori* | SilkDB | DNA | 480,776 | 61 / 138 | 0 / 0 |  |
|  |  |  |  |  |  |  | EST | 101,310 | 1 / 6 | 0 / 0 |  |
|  |  |  | Sphingidae |  | *Manduca sexta* | GenBank | | 419,073 | 34 / 37 | 1 / 1 | 1 |
|  |  |  | all other species | | | GenBank | | 20,329 | 2 / 2 | 0 / 0 |  |
|  |  | Geometroidea |  |  | all species | GenBank | | 23,332 | 0 / 0 | 0 / 0 |  |
|  |  | Noctuoidea | Noctuidae | Noctuinae | *Agrotis segetum* | GenBank | | 45,698 | 4 / 4 | 0 / 2 | 0 |
|  |  |  |  |  | *Athetis lepigone* | GenBank | | 99,385 | 2 / 2 | 2 / 7 | 0 |
|  |  |  |  |  | *Helicoverpa armigera* | GenBank | DNA | 46,057 | 1 / 0 | 1 / 7 | 0 |
|  |  |  |  |  |  |  | EST | 17,684 | 0 / 0 | 0 / 1 | 0 |
|  |  |  |  |  | *Helicoverpa assulta* | GenBank | | 56,052 | 3 / 6 | 2 / 5 | 0 |
|  |  |  |  |  | *Striacosta albicosta* | GenBank | | 4,978 | 0 / 0 | 0 / 1 | 0 |
|  |  |  |  | Amphipyrinae | *Spodoptera exigua* | GenBank | | 166,596 | 6 / 19 | 6 / 12 | 0 |
|  |  |  |  |  | *Spodoptera litura* | GenBank | | 20,578 | 3 / 4 | 2 / 2 | 1 |
|  |  |  |  | Plusiinae | *Trichoplusia ni* | GenBank | DNA | 60,471 | 3 / 14 | 1 / 2 | 0 |
|  |  |  |  |  |  |  | EST | 7,845 | 0 / 0 | 0 / 0 |  |
|  |  |  | all other species | | | GenBank | | 89,698 | 3 / 2 | 0 / 0 |  |
|  |  | Papilionoidea | Nymphalidae | Danainae | *Danaus plexippus* | GenBank | | 474,698 | 17 / 12 | 3 / 5 | 0 |
|  |  |  |  | Heliconiinae | *Heliconius melpomene* | Ensemble Metazoa | DNA | 273,786 | 28 / 34 | 34 / 32 | 8 |
|  |  |  |  |  |  |  | EST | 3,258 | 0 / 0 | 0 / 0 |  |
|  |  |  |  |  | *Heliconius erato* | GenBank | | 7,225 | 2 / 0 | 4 / 4 | 0 |
|  |  |  |  |  | *Heliconius numata* | GenBank | | 3,833 | 0 / 0 | 0 / 1 | 0 |
|  |  |  |  | Nymphalinae | *Melitaea cinxia* | GenBank | | 379,508 | 12 / 12 | 35 / 43 | 1 |
|  |  |  |  | Satyrinae | *Pararge aegeria* | GenBank | | 10,771 | 0 / 0 | 0 / 0 |  |
|  |  |  | Papilionidae |  | *Papilio zelicaon* | GenBank | | 16,621 | 0 / 0 | 0 / 1 | 0 |
|  |  |  |  |  | *Papilio polytes* | GenBank | | 228,669 | 10 / 15 | 0 / 1 | 0 |
|  |  |  |  |  | *Parides eurimedes* | GenBank | | 52,073 | 4 / 17 | 0 / 0 |  |
|  |  |  | Lycaenidae | Polyommatinae | *Polyommatus icarus* | GenBank | | 43,475 | 4 / 11 | 3 / 3 | 1 |
|  |  |  | all other species | | | GenBank | | 306,376 | 22 / 32 | 0 / 0 |  |
|  |  | Pyraloidea | Crambidae | Crambinae | *Chilo suppressalis* | GenBank | | 32,380 | 0 / 3 | 0 / 3 | 0 |
|  |  |  |  | Pyraustinae | *Ostrinia furnacalis* | GenBank | | 46,647 | 7 / 11 | 0 / 0 |  |
|  |  |  |  |  | *Ostrinia nubilalis* | GenBank | DNA | 55,229 | 1 / 4 | 1 / 1 | 1 |
|  |  |  |  |  |  |  | EST | 8,443 | 0 / 0 | 0 / 0 |  |
|  |  |  | Pyralidae |  | *Plodia interpunctella* | GenBank | DNA | 382,059 | 11 / 20 | 2 / 2 | 1 |
|  |  |  |  |  |  |  | EST | 2,956 | 0 / 0 | 0 / 0 |  |
|  |  |  | all other species | | | GenBank | | 27,826 | 0 / 0 | 0 / 0 |  |
|  |  | Tineoidea |  |  | all species | GenBank | | 5,117 | 0 / 0 | 0 / 0 |  |
|  |  | Yponomeutoidea | Plutellidae |  | *Plutella xylostella* | DBM-DB | DNA | 394,063 | 123 / 56 | 101 / 82 | 26 |
|  |  |  |  |  |  |  | EST | 93,618 | 9 / 13 | 4 / 5 | 3 |
|  |  |  | Yponomeutidae |  | *Yponomeuta evonymellus* | GenBank | | 57,817 | 1 / 6 | 2 / 3 | 2 |
|  |  |  | all other species | | | GenBank | | 1,641 | 0 / 0 | 0 / 0 |  |
|  |  | Incurvarioidea |  |  | *Nemophora degeerella* | GenBank | | 51,403 | 4 / 2 | 0 / 0 |  |
|  |  |  | all other species | | | GenBank | | 2,143 | 0 / 0 | 0 / 0 |  |
|  | Zeugloptera |  |  |  | *Micropterix calthella* | GenBank | | 61,201 | 6 / 15 | 0 / 0 |  |
|  |  | all other species | | | | GenBank | | 162 | 0 / 0 | 0 / 0 |  |
|  | all other lepidopteran superfamilies | | | | | GenBank | | 87,588 | 0 / 0 | 0 / 0 |  |
| Tricho­ptera | Annulipalpia |  |  |  | *Philopotamus ludificatus* | GenBank | | 13,993 | 0 / 0 | 0 / 0 |  |
|  | Integripalpia |  |  |  | *Platycentropus radiatus* | GenBank | | 41,264 | 5 / 2 | 0 / 0 |  |
|  | Spicipalpia | Hydroptiloidea |  |  | *Hydroptila* sp. AD-2013 | GenBank | | 42,319 | 1 / 3 | 0 / 0 |  |
|  |  | Rhyacophiloidea |  |  | *Rhyacophila fasciata* | GenBank | | 30,108 | 0 / 0 | 0 / 0 |  |
| Diptera | Brachycera | Asiloidea |  |  | *Bombylius major* | GenBank | | 33,771 | 0 / 0 | 0 / 0 |  |
|  |  | Platypezoidea |  |  | *Megaselia scalaris* | Ensemble Metazoa | | 490,072 | 0 / 0 | 0 / 0 |  |
|  |  |  |  |  |  | GenBank | | 489,467 | 0 / 1 | 0 / 0 |  |
|  |  | Carnoidea |  |  | *Lipara lucens* | GenBank | | 57,469 | 0 / 0 | 0 / 0 |  |
|  |  | Diopsoidea |  |  | *Teleopsis dalmanni* | GenBank | | 180,764 | 9 / 5 | 0 / 0 |  |
|  |  |  |  |  | *Teleopsis whitei* | GenBank | | 65,135 | 0 / 0 | 0 / 0 |  |
|  |  | Ephydroidea |  |  | *Drosophyla melanogaster* | Ensemble Metazoa | DNA | 143,726 | 95 / 41 | 0 / 0 |  |
|  |  |  |  |  |  |  | EST | 60,198 | 0 / 0 | 0 / 0 |  |
|  |  | Sciomyzoidea |  |  | *Themira biloba* | GenBank | | 32,527 | 0 / 0 | 0 / 0 |  |
|  |  | Tephritoidea | Tephritidae | Sepsidae | *Bactrocera dorsalis* | GenBank | | 528,440 | 9 / 1 | 0 / 6 | 0 |
|  |  |  |  |  | *Bactrocera minax* | GenBank | | 38,767 | 0 / 0 | 0 / 0 |  |
|  |  |  |  |  | *Bactrocera cucurbitae* | GenBank | | 851,259 | 49 / 4 | 0 / 0 |  |
|  |  |  |  | Dacinae | *Rhagoletis pomonella* | GenBank | | 8,899 | 0 / 0 | 0 / 0 |  |
|  |  | Muscoidea | Anthomyiidae |  | *Delia antiqua* | GenBank | | 18,110 | 0 / 0 | 0 / 0 |  |
|  |  |  | Muscidae |  | *Musca domestica* | GenBank | | 1,610,683 | 0 / 0 | 0 / 0 |  |
|  |  | Oestroidea | Sarcophagidae |  | *Sarcophaga crassipalpis* | GenBank | | 10,418 | 0 / 0 | 0 / 0 |  |
|  |  |  | Tachinidae |  | *Triarthria setipennis* | GenBank | | 45,913 | 0 / 0 | 0 / 0 |  |
|  | Nematocera | Bibionoidea |  |  | *Bibio marci* | GenBank | | 25,762 | 0 / 0 | 0 / 0 |  |
|  |  | Sciaroidea |  |  | *Sitodiplosis mosellana* | GenBank | | 30,533 | 0 / 0 | 0 / 0 |  |
|  |  | Chironomoidea | Ceratopogonidae |  | *Culicoides sonorensis* | GenBank | | 26,011 | 0 / 0 | 0 / 0 |  |
|  |  |  | Chironomidae | Chironominae | *Chironomus tentans* | GenBank | | 213,463 | 0 / 0 | 0 / 0 |  |
|  |  |  |  | Orthocladiinae | *Belgica antarctica* | GenBank | | 19,115 | 0 / 0 | 0 / 0 |  |
|  |  | Culicoidea | Culicidae | Anophelinae | *Anopheles maculipennis* group | GenBank | | 240,379 | 0 / 0 | 0 / 0 |  |
|  |  |  |  |  | *Anopheles hyrcanus* group | GenBank | | 615,858 | 0 / 0 | 0 / 0 |  |
|  |  |  |  |  | *Anopheles funestus* group | GenBank | | 249,581 | 2 / 2 | 0 / 0 |  |
|  |  |  |  |  | *Anopheles christyi* | GenBank | | 172,664 | 1 / 0 | 0 / 0 |  |
|  |  |  |  |  | *Anopheles gambiae* | Ensemble Metazoa | DNA | 273,109 | 1 / 0 | 0 / 0 |  |
|  |  |  |  |  |  |  | EST | 23,739 | 0 / 0 | 0 / 0 |  |
|  |  |  |  | Culicinae | *Aedes aegypti* | Ensemble Metazoa | DNA | 1,383,974 | 250 / 120 | 0 / 1 | 0 |
|  |  |  |  |  |  |  | EST | 24,177 | 0 / 0 | 0 / 0 |  |
|  |  |  |  |  | *Aedes albopictus* | GenBank | | 70,158 | 0 / 0 | 0 / 0 |  |
|  |  |  |  |  | *Armigeres subalbatus* | GenBank | | 5,463 | 0 / 0 | 0 / 0 |  |
|  |  |  |  |  | *Culex quinquefasciatus* | Ensemble Metazoa | DNA | 579,058 | 8 / 3 | 0 / 0 |  |
|  |  |  |  |  |  |  | EST | 25,621 | 0 / 0 | 0 / 0 |  |
|  |  | Psychodoidea |  |  | *Phlebotomus papatasi* | GenBank | | 251,815 | 1 / 0 | 0 / 0 |  |
|  |  | Trichoceroidea |  |  | *Trichocera saltator* | GenBank | | 71,459 | 0 / 0 | 0 / 0 |  |
|  |  | Tipuloidea |  |  | *Tipula maxima* | GenBank | | 14,879 | 1 / 1 | 0 / 0 |  |
| Hymenoptera | Apocrita | Apoidea | Apidae | Anthophorinae | *Centris flavifrons* | GenBank | | 18,043 | 0 / 0 | 0 / 1 | 0 |
|  |  |  |  | Apinae | *Apis cerana* | GenBank | | 30,730 | 0 / 0 | 0 / 0 |  |
|  |  |  |  |  | *Apis dorsata* | GenBank | | 506,331 | 2 / 0 | 0 / 0 |  |
|  |  |  |  |  | *Apis florae* | GenBank | | 517,573 | 2 / 0 | 0 / 0 |  |
|  |  |  |  |  | *Apis mellifera* | GenBank | DNA | 1,276,747 | 2 / 0 | 0 / 0 |  |
|  |  |  |  |  |  |  | EST | 87,405 | 0 / 0 | 0 / 0 |  |
|  |  |  |  | Bombinae | *Bombus terrestris* | GenBank | DNA | 1,175,479 | 101 / 9 | 0 / 0 |  |
|  |  |  |  |  |  |  | EST | 19,476 | 1 / 0 | 0 / 0 |  |
|  |  |  |  |  | *Bombus insularis* | GenBank | | 16,039 | 6 / 3 | 0 / 0 |  |
|  |  |  |  |  | *Bombus impatiens* | GenBank | DNA | 576,254 | 60 / 23 | 0 / 0 |  |
|  |  |  |  |  |  | BeeBase | EST | 22,455 | 8 / 6 | 0 / 0 |  |
|  |  |  |  |  | *Euglossa cordata* | GenBank | | 14,706 | 0 / 0 | 0 / 0 |  |
|  |  |  |  |  | *Eulaema nigrita* | GenBank | | 15,805 | 0 / 0 | 0 / 0 |  |
|  |  |  |  | Meliponinae | *Frieseomelitta varia* | GenBank | | 9,937 | 0 / 0 | 0 / 0 |  |
|  |  |  |  |  | *Melipona quadrifasciata* | GenBank | | 13,216 | 0 / 0 | 0 / 0 |  |
|  |  |  |  | Xylocopinae | *Exoneura robusta* | GenBank | | 20,073 | 0 / 0 | 0 / 0 |  |
|  |  |  | Halictidae |  | *Megalopta genalis* | GenBank | | 7,384 | 0 / 0 | 0 / 0 |  |
|  |  |  | Megachilidae |  | *Megachile rotundata* | GenBank | | 644,624 | 11 / 13 | 2 / 0 | 0 |
|  |  |  |  |  | *Osmia cornuta* | GenBank | | 64,648 | 1 / 6 | 0 / 0 |  |
|  |  | Chrysidoidea |  |  | *Argochrysis armilla* | GenBank | | 84,952 | 0 / 0 | 0 / 0 |  |
|  |  |  |  |  | *Chrysis viridula* | GenBank | | 50,632 | 0 / 0 | 0 / 0 |  |
|  |  | Sphecoidea |  |  | *Sceliphron caementarium* | GenBank | | 150,433 | 0 / 0 | 0 / 0 |  |
|  |  | Vespoidea | Bradynobaenidae |  | *Chyphotes mellipes* | GenBank | | 129,729 | 0 / 0 | 0 / 0 |  |
|  |  |  | Formicidae | Amblyoponinae | *Stigmatomma oregonense* | GenBank | | 70,047 | 0 / 0 | 0 / 0 |  |
|  |  |  |  | Cerapachyinae | *Cerapachys biroi* | GenBank | | 513,394 | 6 / 6 | 0 / 0 |  |
|  |  |  |  | Dolichoderinae | *Linepithema humile* | GenBank | | 513,121 | 61 / 46 | 0 / 0 |  |
|  |  |  |  | Formicinae | *Camponotus floridanus* | GenBank | | 521,851 | 27 / 16 | 0 / 0 |  |
|  |  |  |  |  | *Nylanderia pubens* | GenBank | | 28,382 | 0 / 0 | 0 / 0 |  |
|  |  |  |  | Myrmicinae | *Acromyrmex echinatior* | GenBank | | 647,617 | 14 / 12 | 0 / 0 |  |
|  |  |  |  |  | *Atta cephalotes* | GenBank | | 646,115 | 4 / 4 | 0 / 0 |  |
|  |  |  |  |  | *Wasmannia auropunctata* | GenBank | | 717,106 | 23 / 12 | 0 / 0 |  |
|  |  |  |  |  | *Pogonomyrmex barbatus* | GenBank | | 516,760 | 7 / 4 | 0 / 0 |  |
|  |  |  |  |  | *Monomorium pharaonis* | GenBank | | 718,913 | 7 / 6 | 0 / 0 |  |
|  |  |  |  |  | *Solenopsis invicta* | GenBank | DNA | 845,469 | 40 / 30 | 0 / 0 |  |
|  |  |  |  |  |  |  | EST | 12,107 | 0 / 0 | 0 / 0 |  |
|  |  |  |  |  | *Vollenhovia emeryi* | GenBank | | 657,444 | 4 / 0 | 0 / 0 |  |
|  |  |  |  |  | *Tetramorium bicarinatum* | GenBank | | 68,526 | 2 / 2 | 0 / 0 |  |
|  |  |  |  | Ponerinae | *Harpegnathos saltator* | GenBank | | 643,879 | 29 / 14 | 0 / 0 |  |
|  |  |  | Mutillidae |  | *Sphaeropthalma orestes* | GenBank | | 78,579 | 0 / 0 | 0 / 0 |  |
|  |  |  | Pompilidae |  | *Pepsis grossa* | GenBank | | 141,419 | 0 / 0 | 0 / 0 |  |
|  |  |  | Scoliidae |  | *Crioscolia alcione* | GenBank | | 205,680 | 0 / 0 | 0 / 0 |  |
|  |  |  | Tiphiidae |  | *Brachycistis timberlakei* | GenBank | | 77,598 | 0 / 0 | 0 / 0 |  |
|  |  |  | Vespidae | Masarinae | *Pseudomasaris vespoides* | GenBank | | 97,230 | 0 / 0 | 0 / 0 |  |
|  |  |  |  | Polistinae | *Mischocyttarus flavitarsis* | GenBank | | 71,187 | 7 / 0 | 0 / 0 |  |
|  |  |  |  |  | *Polistes canadensis* | GenBank | | 67,891 | 8 / 0 | 0 / 0 |  |
|  |  |  |  |  | *Polistes metricus* | GenBank | | 67,891 | 0 / 0 | 0 / 0 |  |
|  |  | Chaldicoidea | Agaonidae |  | *Ceratosolen solmsi* | GenBank | | 578,271 | 0 / 0 | 0 / 0 |  |
|  |  |  | Encyrtidae |  | *Copidosoma floridanum* | GenBank | | 562,386 | 15 / 8 | 0 / 0 |  |
|  |  |  | Pteromalidae |  | *Nasonia giraulti* | GenBank | DNA | 385,864 | 3 / 2 | 0 / 0 |  |
|  |  |  |  |  |  |  | EST | 18,838 | 0 / 0 | 0 / 0 |  |
|  |  |  |  |  | *Nasonia vitripennis* | Ensemble Metazoa | DNA | 888,490 | 43 / 2 | 0 / 0 |  |
|  |  |  |  |  |  |  | EST | 97,707 | 2 / 0 | 0 / 0 |  |
|  |  | Cynipo–idea |  |  | *Ganaspis* sp. G1 | GenBank | | 41,320 | 2 / 7 | 0 / 0 |  |
|  |  |  |  |  | *Leptopilina boulardi* | GenBank | | 30,449 | 0 / 0 | 0 / 0 |  |
|  |  |  |  |  | *Leptopilina clavipes* | GenBank | | 36,464 | 0 / 0 | 0 / 0 |  |
|  |  |  |  |  | *Leptopilina heterotoma* | GenBank | | 36,464 | 5 / 10 | 0 / 0 |  |
|  |  | Ichneumonoidea | Braconidae | Alysiinae | *Asobara tabida* | GenBank | EST | 17,460 | 1 / 0 | 0 / 0 |  |
|  |  |  |  | Microgastrinae | *Cotesia vestalis* |  | | 67,880 | 2 / 2 | 0 / 0 |  |
|  |  |  |  |  | *Microplitis demolitor* | GenBank | | 250,525 | 54 / 16 | 0 / 0 |  |
|  |  |  |  | Opiinae | *Fopius arisanus* | GenBank | | 413,967 | 92 / 6 | 0 / 0 |  |
|  |  | Platygastroidea |  |  | *Telenomus podisi* | GenBank | | 55,042 | 0 / 0 | 0 / 0 |  |
|  |  | Orussoidea |  |  | *Orussus abietinus* | GenBank | | 532,483 | 59 / 51 | 0 / 0 |  |
|  |  | Tenthredinoidea | Tenthredinidae | Allantinae | *Athalia rosae* | GenBank | | 389,017 | 2 / 4 | 0 / 0 |  |
|  |  |  |  | Tenthrediniinae | *Tenthredo koehleri* | GenBank | | 54,532 | 0 / 0 | 0 / 0 |  |

GenBank taxonomy was used. Sequence sources: GenBank (<http://www.ncbi.nlm.nih.gov/genbank>), SilkDB ([http://www.silkdb.org](http://www.silkdb.org/)), WildSilkbase ([http://www.cdfd.org.in](http://www.cdfd.org.in/)), EnsembleMetazoa ([http://metazoa.ensembl.org](http://metazoa.ensembl.org/)), DBM-DB (<http://iae.fafu.edu.cn/DBM/>), and BeeBase: (<http://hymenopteragenome.org/beebase/>).

**Supplementary References**

Finn, R. D., Bateman, A., Clements, J., Coggill, P., Eberhardt, R. Y., Eddy, S. R., Heger, A., Hetherington, K., Holm, L., Mistry, J., Sonnhammer, E. L., Tate, J., and Punta, M. (2014). Pfam: the protein families database. *Nucleic Acids Res*. 42, D222-D230. doi: 10.1093/nar/gkt1223.

Malik, H. S., Burke, W. D., and Eickbush, T. H. (1999). The age and evolution of non-LTR retrotransposable elements. *Mol. Biol. Evol.* 16, 793-805.

Pearson, W. R. and Lipman, D. J. (1988). Improved tools for biological sequence comparison. *Proc. Natl. Acad. Sci. USA* 85, 2444-2448.

Solovyev, A. G., Minina, E. A., Makarova, S. S., Erokhina, T. N., Makarov, V. V., Kaplan, I. B., Kopertekh, L., Schiemann, J., Richert-Pöggeler, K. R., and Morozov, S. Y. (2013). Subcellular localization and self-interaction of plant-specific Nt-4/1 protein. *Biochimie* 95, 1360-1370. doi: 10.1016/j.biochi.2013.02.015.

Thompson, J. D., Higgins, D. G., and Gibson, T. J. (1994). CLUSTAL W: improving the sensitivity of progressive multiple sequence alignment through sequence weighting, position-specific gap penalties and weight matrix choice. *Nucleic Acids Res*. 22, 4673-4680.

Wahlberg, N., Wheat, C. W., and Peña, C. (2013). Timing and patterns in the taxonomic diversification of Lepidoptera (butterflies and moths). *PLoS ONE* 8, e80875. doi: 10.1371/journal.pone.0080875.
